# Improving Schools’ Readiness for Involvement in Suicide Prevention: An Evaluation of the Creating Suicide Safety in Schools (CSSS) Workshop

**DOI:** 10.3390/ijerph16122165

**Published:** 2019-06-19

**Authors:** Patricia Breux, Dana E. Boccio

**Affiliations:** 1 Suicide Prevention Center of New York, 150 Broadway, Suite 301, Menands, NY 12204, USA; pat.breux@omh.ny.gov; 2 Dana E. Boccio, Derner School of Psychology, Adelphi University, 1 South Avenue, Garden City, NY 11530, USA

**Keywords:** youth suicide, school-based suicide prevention, training, workshop, barriers

## Abstract

Schools have an important role to play in combatting suicide, a significant public health problem that disproportionately affects adolescents and young adults. Schools can work to reduce youth suicidality by adopting policies that align with best practice recommendations pertaining to suicide prevention, intervention, and postvention. This study examined the impact of a one-day training, the Creating Suicide Safety in Schools (CSSS) workshop, on the readiness of school personnel to improve their schools’ suicide-related policies and procedures. Participants (*N* = 562) consisted predominantly of school-based mental health professionals working in communities of low or mixed socioeconomic status in New York State. Survey data were collected according to a one-group pre-test—post-test design with a 3-month follow-up. Workshop participants demonstrated improvements from pre-test to post-test in their attitudes about the importance of school-based suicide prevention, knowledge of best practices, perceptions of administrative support, and feelings of empowerment to work collaboratively to enhance their schools’ suicide safety. At follow-up, participants reported barriers to implementing changes, most commonly in the form of insufficient time and stigma surrounding the topic of suicide. The results of this study provide preliminary evidence for the effectiveness of the CSSS workshop as a promising method for improving schools’ suicide safety, yet additional research using randomized controlled trials needs to be conducted.

## 1. Introduction

Suicide constitutes a serious public health problem that disproportionally affects young people in the US and worldwide [1]. Globally, suicide is the second leading cause of mortality among 15- to 29-year-olds [2]. According to the most recent data reported by the Centers for Disease Control and Prevention, while suicide was responsible for fewer than 2.3% of deaths within the entire US population in 2016, it accounted for 20.2% of the deaths of young people aged 15 to 24 years [3]. Moreover, data from the Youth Risk Behavior Surveillance System (YRBSS), a large-scale survey of US high school students administered biennially, reveal that a considerable minority of youth experiences some degree of suicidality [4]. Specifically, during the 12 months preceding the survey, 17.2% of students in grades 9–12 seriously considered attempting suicide, 13.6% made a suicide plan, 7.4% engaged in at least one suicide attempt, and 2.4% made an attempt that was responded to with medical attention. Despite state, national, and international efforts to combat this significant public health threat, youth suicide has been on the rise [5,6].

Addressing the problem of youth suicide requires collaborative and synergistic action across various community institutions and agencies, a position that has been codified in key documents informing suicide prevention initiatives. The World Health Organization’s [7] *Preventing Suicide: A Global Imperative* recommends that policy-makers and relevant stakeholders adopt a “comprehensive multisectoral strategy” (p. 2) to advance a public health agenda that prioritizes suicide prevention. Similarly, the US Department of Health and Human Services’ [8] *National Strategy for Suicide Prevention* advocates for greater involvement of both public and private sectors in suicide prevention initiatives and enumerates several settings deemed appropriate for the integration of suicide prevention activities into existing programs and services. The education system has gained recognition as a logical venue for the furtherance of suicide prevention efforts [9], and schools have assumed more of a leadership role in identifying, referring, and providing assistance to youth with mental health needs [10]. Kalafat [11] noted that schools are responsible for the education, socialization, and protection of youth; thus, activities related to suicide prevention are compatible with a school system’s traditional mandates and mission. Moreover, positive and caring school environments that foster a sense of interpersonal connectedness, encourage disclosure of concerns to supportive adults, and cultivate competencies associated with psychological wellness can serve as a protective function that mitigates suicide risk [12].

Suicide prevention initiatives have been embedded within the multitiered systems of support (MTSS) paradigm that structures the delivery of academic and behavioral interventions in schools [13]. Efforts to prevent suicide and reduce suicide risk within an MTSS framework mirror a public health approach to suicide prevention, with interventions organized according to three different levels or tiers: universal (Tier 1), selected (Tier 2), and indicated (Tier 3). Universal (Tier 1) programs are delivered to the entire school population and often take the form of gatekeeper trainings for school personnel and/or students [9,14]. These programs disseminate information about risk factors and warning signs, dispel myths about suicide, promote help-seeking behavior, and teach how to respond effectively to an at-risk student and connect that individual to appropriate resources. Upstream suicide prevention approaches that foster resilience and attenuate the emergence of mental health problems and suicidality are also considered universal-level strategies [15]. These initiatives encompass system-level interventions that establish a positive and nurturing school climate, promote prosocial norms, and strengthen students’ self-regulatory and coping mechanisms through curricula that build social–emotional competencies such as problem-solving, conflict resolution, and distress tolerance skills [12]. Universal-level approaches comprise the majority of school-based suicide prevention programs that have been systematically evaluated [14]. Several programs have demonstrated effectiveness in enhancing the responsiveness of gatekeepers and improving knowledge and attitudes related to suicide and help-seeking [16,17,18,19]. However, only the Good Behavior Game [20], the Signs of Suicide (SOS) program [21,22], and the Youth Aware of Mental Health Programme (YAM) [23] have been shown to reduce suicidal behavior [13,24]. This dearth of evidence has been attributed to methodological shortcomings that are often inherent to suicide prevention research, such as the low base rate of suicide in the general student population and the challenges associated with instituting control conditions, ensuring implementation fidelity, and assessing distal behavioral outcomes [9,11].

Selected (Tier 2) suicide prevention programs target subpopulations of students who have been exposed to certain epidemiologically-established risk factors (e.g., family dysfunction, delinquency, academic difficulties) that contribute to the expression of suicidal behavior. At this level, targeted screenings and comprehensive risk assessments may be conducted to identify students in need of additional support [25]. Individuals who are considered to be at risk may be provided with psychoeducational interventions within the school, as well as referrals to community-based service providers [13]. Indicated (Tier 3) approaches are characterized by more intensive support and are directed toward students who self-identify or who are identified by others as high risk due to the presence of suicidal thoughts, plans, and/or behaviors. Activities at this level are aimed at ensuring student safety, minimizing distress, and treating underlying psychopathology. Tier 3 interventions include safety planning; communication among school personnel, parents, and outside mental health service providers; counseling/psychotherapy; possible hospitalization; facilitation of school reentry; ongoing monitoring of suicide risk; and postvention in the aftermath of a suicide [13,26].

The empirical literature has generally converged on the conclusion that there is value to educating school personnel about the signs of suicide and how to assist students who are at risk [19]. Gatekeeper training programs have been commonly adopted by schools as a means of instructing school staff in how to identify suicidal students and link them to appropriate resources and services [27,28]. The premise underlying the development of school-based gatekeeper training programs is that youth struggling with suicidality often do not seek help from adults and, thus, are under-identified [29]. It stands to reason that by training teachers, counselors, administrators, and other school personnel in the risk factors and warning signs associated with suicidality, and by increasing staff’s self-efficacy related to assisting at-risk youth, a larger number of suicidal students will be guided toward some form of mental health intervention.

Studies examining the effectiveness of school-based gatekeeper programs have generally determined that this approach results in increased knowledge about suicide, improved attitudes toward involvement in suicide prevention, and greater self-efficacy/confidence to intervene in order to assist a suicidal student [18,30,31]. However, there has been less of a focus in the literature on the translation of these enhanced self-appraisals into behavioral outcomes, such as gatekeeper skills (e.g., asking students about suicide, comfortably communicating with students in crisis), referral behaviors, and student suicide and suicide attempts. Wyman and colleagues [31] conducted the first randomized controlled trial of a widely implemented gatekeeper training program entitled, Question, Persuade, Refer (QPR) [32], using secondary school personnel. Findings supported the overall effectiveness of QPR in improving school staff’s self-reported knowledge and perceptions of efficacy and preparedness to perform a gatekeeper role. However, behavioral change, in the form of increased queries about suicide, was restricted to individuals who were already communicating with distressed youth. Specifically, participants who demonstrated increases in the frequency with which they asked students about suicide were those school personnel who reported greater involvement in discussing suicide and other emotionally-laden issues with students prior to the training. Cross et al. [33] found that adding a behavioral rehearsal component (i.e., role play practice) to traditional gatekeeper training resulted in improved gatekeeper skills (e.g., use of direct questions about suicide, active listening, clarifying questions), as rated by independent observers. In general, research largely supports the provision of training to school personnel as a promising component of school-based suicide prevention. Nevertheless, it is recommended that such programs be integrated into a broader and more comprehensive suicide prevention approach [24,34].

Cooper, Clements, and Holt [34] advocate for the adoption of “hybrid programs” (p. 701) that integrate gatekeeper training, screening, and curricular elements. School personnel may be heartened by the existence of turnkey, commercially available programs and public domain products to guide the implementation of suicide prevention initiatives (for example, see the Suicide Prevention Resource Center’s website on resources and programs at http://www.sprc.org/resources-programs). Nevertheless, school administrators and staff may find themselves confused and overwhelmed by the number of program options, variability in components, and limited evidence pertaining to program effectiveness in reducing youth suicide and suicidal behavior. Moreover, program selection and implementation are inevitably influenced by organizational factors, such as administrative support, stakeholder buy-in, availability of on- and off-campus resources, the culture of a particular school or district, and the values of the surrounding community [35,36]. Consideration of these ecological and logistical variables when adopting, developing, or adapting a particular program is critical to successful suicide prevention and may constitute the difference between prevention efforts that are performed in a perfunctory manner and those that are embraced with enthusiasm and zeal.

School administrators and staff will likely need guidance in determining which approach addresses—or can be tailored to address—the particular needs of their building or district. The Creating Suicide Safety in Schools (CSSS) workshop was designed to “meet schools where they are” by encouraging school personnel to examine their current practices related to suicide prevention, response, and postvention and bring them into alignment with best practices. While incorporating information related to youth suicide, risk factors, and warning signs, the CSSS workshop ventures beyond traditional gatekeeper programs to assist school personnel in creating a roadmap for improving suicide safety in their respective school settings. Thus, by focusing on schools’ readiness for involvement in suicide prevention, the basic framework of the CSSS workshop can be differentiated from the structure and purpose central to conventional gatekeeper trainings, which tend to have a more narrow emphasis on educating school personnel about the signs of suicide and how to make referrals.

The CSSS workshop is a one-day interactive training developed to engage school-based multidisciplinary teams in a process to (1) evaluate their own schools’ existing suicide prevention and intervention readiness, (2) receive evidence-based and best practice guidance, (3) develop a school-specific comprehensive suicide prevention and response plan, and (4) learn about resources to enhance the safety and health of a school environment that are subsidized or available at low or no cost. The workshop was developed by Pat Breux at the Suicide Prevention Center of New York in conjunction with the New York State Office of Mental Health (OMH) and has been offered in the NY area for over 6 years. It incorporates concepts consistent with a public health prevention model, highlighting opportunities for intervention according to an MTSS framework. Drawing from risk/resiliency theory [37] and ecological models of development (e.g., [38]), a youth’s risk of suicide is conceptualized as resulting from complex interactions between suicidogenic and protective factors that exist along a socioecological continuum (i.e., occurring at the individual, relationship or environmental level). Schools are encouraged to direct their prevention efforts at reducing or eliminating risk factors for suicide and increasing protective factors, particularly at the environmental level. Central to this approach is the establishment of knowledgeable and nurturing school communities that emphasize shared responsibility for safety and well-being (see [39] for a more detailed explanation).

The present study constitutes an evaluation of the effectiveness of the CSSS training in increasing workshop participants’ readiness to move forward with efforts to enhance their schools’ suicide safety policies and procedures. The purpose of the current investigation was to determine the impact of the CSSS workshop on participants’ (1) attitudes about the importance of suicide prevention and schools’ roles in such efforts, (2) knowledge of best practices for suicide prevention/intervention and familiarity with resources for advancing suicide safety, (3) perceptions regarding the provision of administrative support for enacting suicide safety measures, and (4) feelings of empowerment to improve schools’ practices related to suicide prevention and response. In addition, participants’ satisfaction with the workshop and their perceptions regarding the most valuable components of the training were examined. Obstacles to improving school-based suicide safety and factors facilitating the adoption of suicide prevention/response initiatives were also explored.

## 2. Materials and Methods

Survey data were collected according to a one-group pre-test–post-test design with a 3-month follow-up assessment. All participants gave their informed consent for inclusion before they participated in the study. The study was conducted in accordance with the Declaration of Helsinki, and the protocol was approved by the Institutional Review Boards of Adelphi University (#100815) and the New York City Department of Education (#1346). Recruitment of workshop attendees involved a dual-pronged strategy. Specifically, advertisements were posted online by the Suicide Prevention Center of New York, and invitations to participate were extended to schools identified as needing assistance by the New York City Office of School Health. A master list was created by the investigators linking each workshop attendee’s name with a unique 3-digit identification number. Upon arriving at the training location, workshop participants were given a name tag that included their first and last names and ID number. Attendees were offered a brief explanation of the research being conducted and provided with the “Pre-Workshop Survey” and informed consent form. They were instructed to complete the survey only if they wished to participate in the study. After completion of the full-day training, attendees were immediately administered the “Post-Workshop Survey” and were asked to provide their contact information on a separate form if they were willing to participate in the follow-up phase of the study. In order to maintain the confidentiality of their responses, participants were instructed to write only their ID numbers on both the pre- and post-workshop survey instruments. Three months after the training, participants who provided their contact information were sent an email containing a link to the follow-up questionnaire. Individuals who did not respond to the initial email request were contacted again one week later with a reminder to complete the online survey.

The CSSS workshop was designed to introduce school personnel to best practice recommendations regarding suicide safety, as well as an array of suicide prevention, intervention, and postvention resources that schools can adopt or integrate into already existing school-based initiatives. While individual school administrators and staff members were welcome to attend the training, schools were encouraged to send “planning teams” consisting of 3 to 5 individuals who would be involved in reviewing and refining their schools’ approach to addressing youth suicide. During the training, school personnel spent time problem solving for specific actions needed to create suicide-safer schools. Workshop attendees were guided through a process of evaluating their settings’ current practices and developing short-term goals in six areas: (1) ensuring appropriate training for staff and faculty, (2) promoting resilience in students, (3) identifying, assessing, and responding to students at risk for suicide, (4) planning for recovery after a suicide loss, (5) engaging and educating parents, and (6) establishing collaborative relationships with community health, mental health, and human service providers. Each school professional was provided with a 339-page binder that contained an extensive collection of public domain resources pertaining to these six areas. Attendees were encouraged to consult these materials during their development of standardized protocols and procedures for assessing and managing suicide risk and reducing the likelihood of contagion after a suicide event. The workshop’s format consisted of a didactic PowerPoint presentation coupled with small workgroup discussions, video clips, case scenarios, checklists, group planning documents, and exposure to free and low-cost materials consistent with best practice recommendations and/or evidence-based practice standards. Participants were urged to share the workshop materials with school staff and administrators who were not in attendance so as to facilitate the adoption of effective practices. Thus, individuals who attended the training returned to their schools/districts with a comprehensive plan for improving suicide safety and specific resources to enable the implementation of this plan.

A review of the literature related to school-based gatekeeper training programs revealed that evaluations of such programs have typically incorporated measures assessing participants’ suicide awareness and knowledge, attitudes toward suicide and self-harm, and self-efficacy and personal competence related to intervening to support a potentially suicidal youth [16]. These measures were either self-developed by program researchers or adapted from instruments used in previous studies. Considering that the CSSS workshop is not a traditional gatekeeper training program and no existing measures were deemed adequate to capture the workshop’s specific objectives, pre-test, post-test, and follow-up measures used in the present study were developed by the investigators to align more precisely with the goals of the CSSS training.

Prior to the commencement of the workshop, participants were asked to complete a 32-item “Pre-Workshop Survey” inquiring about participants’ previous suicide-related training, reasons for attending the workshop, and demographic information. Fifteen items were included to evaluate attendees’ attitudes about suicide and school involvement in suicide prevention, knowledge of best practices and resources for enhancing suicide safety, perceptions of administrative support, and feelings of empowerment to move forward with suicide safety measures. At the conclusion of the workshop, participants were administered a 40-item “Post-Workshop Survey”, which included the same 15 items, as well as questions assessing participants’ satisfaction with the training. Two open-ended questions provided an opportunity for participants to share their impressions regarding the most valuable components of the workshop and any obstacles they anticipated would interfere with the implementation of suicide safety initiatives in their schools. Participants who provided their contact information at post-test were emailed a brief follow-up survey 3 months after completing the training. Questions pertained to schools’ movement toward the adoption of enhanced suicide safety practices, factors that served to aid and impede these advancements, and goals that had yet to be accomplished.

For the pre-test and post-test assessments, composite scores were calculated for “Attitudes”, “Knowledge”, and “Support” by summing participants’ scores on individual items developed to assess each area. Specifically, the Attitude composite score, reflecting attendees’ beliefs about the importance of suicide prevention, was created by adding participants’ responses to items 1 and 2. Internal consistency was good, as indicated by a Cronbach’s α of 0.83 at pre-test and 0.87 at post-test. The Knowledge composite score, representing participants’ knowledge about suicide prevention- and intervention-related best practices and resources, was composed of responses to items 5, 6, 7, 8, and 9 (α = 0.81 at pre-test and α = 0.83 at post-test). Items 3, 4, 10, 11, 12, and 13 comprised the Support composite, a measure of attendees’ perceived levels of administrative support for efforts aimed at improving school-based suicide safety (α = 0.84 at pre-test and α = 0.86 at post-test). A total scale score was also obtained by summing responses to all 15 items. Reliability was considered good, with α = 0.88 and 0.89 for pre-test and post-test assessments, respectively. For all composites, higher scores were in the desired direction and reflected more positive beliefs and attitudes. Cohen’s *d* was calculated to determine the magnitude of the CSSS Workshop’s impact on participants’ attitudes, knowledge, perceptions of support, and overall beliefs/perceptions related to suicide safety. Content analyses were performed on qualitative data obtained from participants’ open-ended responses to questions included at post-test. Responses were coded for themes and frequency by two reviewers (school psychology graduate students), and any disagreement was resolved through discussion with a third reviewer (faculty member), resulting in consensus on thematic categorization.

**Figure 1:**
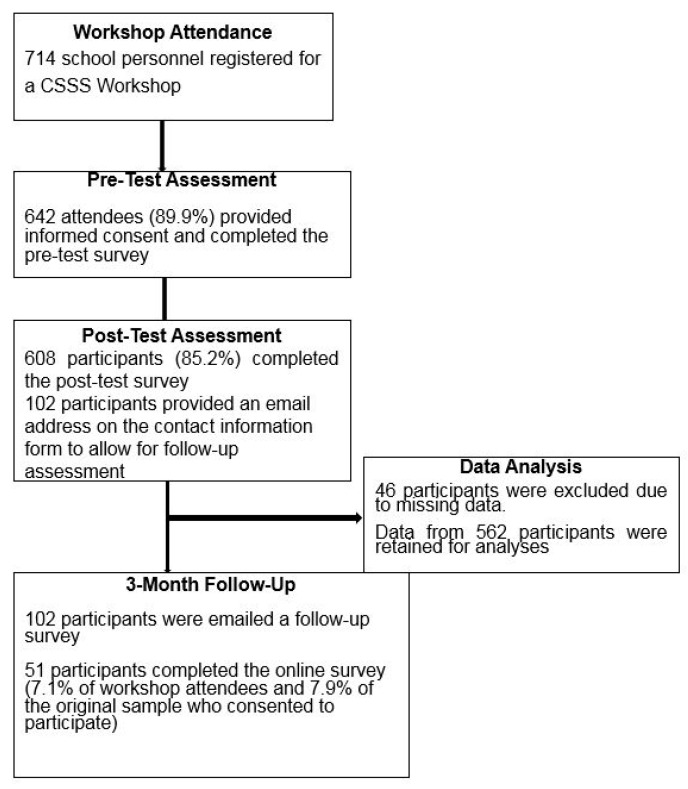
Flowchart of workshop attendees who participated in the investigation.

A total of 714 school personnel registered to attend one of the scheduled CSSS workshops. Of these individuals, 642 (89.9% of attendees) consented to participate in the study and completed the pre-workshop survey. After participating in the workshop, 608 participants (85.2% of attendees) completed the post-test questionnaire. Forty-six participants were excluded from analyses due to missing data, resulting in a final sample of 562 participants. It should be noted that inconsistent procedures were utilized in obtaining contact information from study participants for follow-up assessment. Some workshop attendees were not provided with contact information forms due to oversight on the part of the presenter. Additionally, the investigators learned that some participants decided to nominate one individual from their school to complete the form and serve as the designated respondent at follow-up. In total, 102 participants completed the forms and were contacted 3 months subsequent to the completion of the workshop. Fifty-one individuals responded to the online questionnaire, resulting in a 50% response rate for those who were invited to participate in the follow-up phase of the investigation. Individuals who participated at follow-up represented 7.1% of total workshop attendees and 7.9% of the original sample who consented to participate in the study. Figure 1 summarizes the flow of participants and exclusion/retention of data for analyses.

Participants (*N* = 562) had a mean age of 41.77 years (*SD* = 10.24) and had occupied their current position for an average of 9.97 years (*SD* = 8.46). Attendees were predominantly female (86.1%) and worked in communities that were primarily of low (44.5%) or mixed (45.5%) socioeconomic status, with medium- and high-status communities represented to a lesser extent (7.7% and 2.3%, respectively). Approximately half of the participants (48.1%) served urban communities, with the remainder divided between rural (26.7%) and suburban (18.7%) areas (6.4% represented some combination). Participants held a total of 63 different job titles. School-based mental health professionals comprised a sizeable portion of participants and consisted largely of school counselors (41.1%), social workers (22.9%), and school psychologists (11.6%). Other professions attended in smaller numbers, including administrators (e.g., principals, assistant superintendents, program coordinators, Directors of Student Services), teachers, nurses, and counseling and social work interns. Most participants had earned either a Master’s degree (28.1%) or a Master’s degree plus additional credits (62.8%), with substantially fewer participants holding a Bachelor’s degree (5.0%), doctorate (2.5%), associate’s degree (1.1%), or high school diploma (0.5%) as their highest level of education. Participants’ employment settings consisted of the following: 28.9% high school, 21.6% elementary school, 15.9% middle school, 29.1% multiple school levels, and 4.5% other (e.g., community agency, mental health department, alternative educational placement). Slightly more than half (55.8%) reported that they had participated previously in suicide prevention-related training and had accrued an average of 9.5 hours (*SD* = 10.49) of such instruction. One in three attendees (33.6%) reported that their school had recently been affected by suicide. Approximately two-thirds (64.9%) of participants attended the workshop with at least one additional individual from their school/district, while 35.1% reported that they were the sole representatives of their school or agency. Comparisons between demographic data obtained from the larger sample (*N* = 562) and characteristics of the sample at follow-up (*n* = 51) revealed no significant differences related to age, gender, years in current position, education level, employment setting, and community’s socioeconomic status.

## 3. Results

Over a period of approximately two years (between November 3, 2015 and December 7, 2017), data were collected from 28 CSSS trainings that were held in the state of New York. After accounting for missing survey responses, data from 562 attendees were deemed suitable for analysis. Note that results from 87 participants included in these analyses were previously reported in Breux, Boccio, and Brodsky [31]. Table 1 presents pre- and post-scores from 15 items related to participants’ attitudes, knowledge, perceptions of administrative support, and feelings of empowerment to work collaboratively with colleagues for the purpose of improving their schools’ suicide safety. All items were rated on a 5-point scale, with 1 equivalent to “Strongly Disagree” and 5 representing “Strongly Agree”. Significant increases in average scores were observed for all items. However, while these gains are statistically significant, some scores did not change in a clinically significant fashion from pre-test to post-test. For example, scores on items 1 and 2 were already high at pre-test, and the increases observed at post-test were relatively modest and not likely to be clinically meaningful.

Table 2 presents average composite scores observed at pre- and post-test, revealing that significant improvements were noted in all areas assessed, with the greatest gains observed in the area of knowledge. Moreover, separate analyses focusing solely on participants who had previously received some form of suicide prevention-related instruction yielded significant increases on the composite scales from pre- to post-workshop. Although the workshop incorporated group-based activities designed to encourage team-building and partnership with others in suicide prevention efforts, individuals who attended the CSSS training alone (as opposed to part of a school- or district-based team) demonstrated significant increases in all areas. Effect sizes obtained were generally medium to large, with changes in knowledge scores yielding the most substantial effects, particularly for participants who lacked prior training in suicide prevention and response.

Table 3 presents participants’ satisfaction ratings regarding the CSSS workshop. Results demonstrated high levels of satisfaction, with the vast majority of attendees having perceived the workshop to have been useful, informative, and relevant to their jobs. Almost all participants indicated that they would recommend the workshop to others.

Overall, 382 participants offered responses to an open-ended item assessing workshop attendees’ perceptions of the most valuable components of the training, resulting in the identification of seven categories: binder with free or low-cost materials/resources (64.4%); information provided, such as risk factors, warning signs, and evidence-based practice recommendations (28.0%); networking/discussion opportunities (7.6%); role-playing/group work (6.5%); presenter expertise (4.5%); protocols and templates (4.2%); and presentation of case scenarios (3.9%). Four hundred and one participants listed obstacles that they believed interfered with the enactment of suicide prevention efforts in their schools. The most frequently identified perceived barriers to implementation of suicide safety and prevention programs included insufficient time and resources, including financial concerns and problems related to understaffing (40.7%), and a lack of support from staff, administration, and parents in the form of apathy, failure to understand the importance of addressing the issue or active resistance (29.4%). Other barriers mentioned were difficulties associated with training staff and securing cooperation from colleagues (10.0%); stigma and discomfort surrounding discussing the topic with students (5.9%); perceived irrelevance of suicide prevention initiatives to elementary school settings (1.7%); inconsistent follow-up with students (1.7%); unclear school procedures for handling suicidality (1.5%); lack of community resources (1.2%); and school staff’s lack of cultural competence (1.0%).

Follow-up data were obtained from 51 participants who completed the online questionnaire. Table 4 presents participants’ perceptions at follow-up regarding their schools’ responsiveness to information and recommendations provided during the workshop and the implementation of changes since the training. Overall, the ideas and resources brought back from the workshop were generally received positively. Forty-four percent of respondents claimed that their schools had made improvements to suicide safety practices since the training, and about half of the participants indicated that the workshop helped to enhance their schools’ efforts in this area. More than a third of the participants revealed that their schools had made progress with respect to responding to at-risk students and preventing suicide. However, there was substantial variability in participants’ responses, suggesting that schools differed in their willingness to embrace and adopt enhanced practices surrounding suicide prevention and response.

Barriers to implementing suicide safety initiatives were also examined at follow-up using a close-ended question with multiple predetermined response options (see Table 5). Participants indicated that the most frequently encountered obstacles were inadequate time to address the problem of youth suicide and the existence of stigma associated with discussion of the topic. Additional challenges were mentioned by 7 participants who selected the “other” response option. These included prioritization of academics over mental health and the school community’s resistance to acknowledging their interconnectedness, lack of leadership and follow-through with programs/procedures, perceptions that this is not a pressing issue at the elementary level, and heavy workloads that render it difficult to act “proactively.”

Nevertheless, reports of progress were also noted in participants’ open-ended responses to an item exploring accomplishments related to suicide safety that had been achieved since the training. Thematic analysis of responses offered by 40 participants indicated that workshop attendees had helped their schools to develop clearer protocols with respect to student referral, risk assessment, and safety planning (52.5%); shared information from the training with teachers, staff, and students (32.5%); and compiled a list of community resources, including contact information for mental health service providers (10.0%). Participants also reported that these changes were facilitated by the garnering of administrative support, the championing of suicide safety initiatives by highly motivated individuals, and the recognition of a compelling student need (e.g., a perceived increase in the number of students experiencing suicidal ideation). Twenty-nine participants provided responses to an open-ended item inquiring about outstanding goals related to suicide safety. Participants generally expressed a need for more (and ongoing) professional development for staff and training for students (62.1%), clearer procedures and greater awareness of safety protocols (17.2%), improved administrative support in terms of the prioritization of suicide prevention by those in leadership positions (13.8%), increased local agency involvement and communication/collaboration across service delivery settings (10.3%), and a stronger focus on social–emotional learning and methods for promoting resiliency (6.9%).

## 4. Discussion

The present study represented a preliminary evaluation of the impact of the Creating Suicide Safety in Schools (CSSS) workshop on attendees’ attitudes about the importance of suicide prevention, knowledge of best practices and available evidence-based resources, perceptions of administrative backing and support, and sense of empowerment to move forward with school-based suicide safety measures. Workshop participants showed significant improvements from pre-test to post-test in all areas assessed. Individuals with and without prior suicide-specific training, and those who attended alone or with others, were similarly likely to demonstrate gains in training outcomes. Moreover, attendees were highly satisfied with their experience and perceived the training to be useful and informative. At the 3-month follow-up, approximately half of participants reported that they believed the workshop had helped to improve their school’s suicide safety. However, these results must be interpreted with caution due to the limited sample size at this assessment time-point. Variability was noted at follow-up with respect to schools’ progress toward adopting enhanced suicide prevention and response practices. These results suggest that some schools will require additional guidance and encouragement beyond that offered during the workshop to move forward with best practice recommendations. They also highlight the potential influence of organizational factors in facilitating or hampering advancements in suicide safety, a finding which is consistent with previous writings advocating for the consideration of environmental variables when implementing school-based suicide prevention measures [35]. For example, participants in this study reported that positive changes to suicide-related practices were driven by the support of administrators and the enthusiasm of a select few individuals who assumed responsibility for promoting these improved procedures. Thus, while the CSSS workshop attempts to build on schools’ current practices and resources, future research might explore how the training might incorporate strategies for generating broader interest in, and ownership of, suicide safety efforts. This would conceivably assist attendees in navigating the challenge of translating best practice recommendations into overt action, which can be particularly difficult in schools characterized by resistance to change.

Qualitative data from open-ended responses at follow-up offered a tentative look at actions adopted by schools since the workshop, as well as areas requiring additional development. The limited data collected revealed that schools most commonly made strides in the clarification of referral procedures, selection of risk assessment tools, and compilation of outside resources. Outstanding goals related to improving suicide safety were identified by participants and provide insight into possible future targets for intervention. These included additional training for staff and students, agreement on clearer suicide safety protocols, and greater collaboration within and across settings. Broader systemic obstacles, such as the hectic schedules of school personnel and the stigma attached to discussing suicide, presented a challenge to schools interested in embracing best practices related to suicide prevention, response, and postvention. The literature on school-based suicide prevention and intervention underscores a reluctance on the part of many schools to adopt certain suicide safety initiatives [40]. According to Erbacher, Singer, and Poland [25], “it often takes multiple student deaths in the same school or district to prompt more focus on suicide prevention” (p. 8). Results from the current study suggest that schools’ timidity in addressing youth suicide may stem from a combination of logistical concerns (e.g., overextended personnel, insufficient resources) and a general discomfort with confronting the issue. These obstacles are not easily overcome, but establishing a more universal set of expectations surrounding school involvement in suicide safety and clearly delineating roles for school personnel could socialize educational institutions into assuming greater responsibility for combatting youth suicide. Perceptions of educators working on the frontlines are especially important to consider when exploring how to counter barriers to suicide prevention efforts in the schools. Research demonstrates that teachers believe they have a role to play in suicide prevention; nevertheless, their comfort and confidence levels pertaining to assisting suicidal students are adversely affected by a lack of training and apprehension surrounding the possibility of making the situation worse and/or experiencing legal repercussions [41]. Training related to identifying and intervening with suicidal youth should include teachers and school staff (e.g., paraprofessionals, nurses, cafeteria workers), while simultaneously not overwhelming these school personnel with responsibilities that venture beyond their skills and expertise. 

The present investigation suffers from certain methodological limitations that constrain the interpretation of results. While significant increases in scores were observed from pre-test to post-test, the lack of a control group precludes the drawing of definitive causal conclusions regarding the impact of the workshop. The immediate nature of the pre/post-test assessment time-points renders it easier to conclude that any changes observed were specific to the training. However, it is not possible to determine conclusively whether the gains observed were a natural consequence of bringing stakeholders together to discuss suicide prevention or the result of content specifically covered during the training. Moreover, inconsistent procedures for collecting contact information resulted in low levels of participation at follow-up, thus rendering it difficult to determine the representativeness of data obtained from participants during this phase. For example, it is possible that greater participation might have resulted in less auspicious findings regarding the enactment of improvements to suicide safety practices after the workshop. Similarly, since only half of respondents who were invited to participate at follow-up completed the survey, it is impossible to rule out the potential effects of response bias—i.e., perhaps those individuals who responded were working in schools that were more receptive to implementing recommendations from the workshop. Thus, conclusions drawn from the interpretation of follow-up data must be regarded as highly tentative. Future research examining the effects of participating in the CSSS workshop should strive to secure more extensive participation at follow-up, as well as obtain quantitative data regarding the specific actions taken by schools after the training (e.g., implementation of school-wide screenings, adoption of clear referral procedures, development of collaborative relationships with hospitals/community agencies). Additional studies should include standardized measures to evaluate pre/post-test changes in the variables under investigation. While existing instruments did not precisely meet the needs of this evaluation, the failure to incorporate standardized measures renders it difficult to compare the impact of this intervention with changes observed for other programs. Future research should also attempt to link changes in the proximal variables included in this investigation (i.e., attitudes, knowledge, perceptions of support, feelings of empowerment) with subsequent modifications to school practices and more distal outcomes, such as suicidal behavior and suicide deaths. The lack of objective measures of change is a weakness of this investigation and is reflective of a broader sector evidence gap.

## 5. Conclusions

The CSSS workshop shows promise as a potentially effective means of promoting suicide safety in schools by improving the attitudes, knowledge, and confidence of school staff in tackling a challenging public health problem. Educational settings can serve as a force for positive change by amplifying the power of protective factors to create a “social ecology of wellness” [42] (p. 246) that is conducive to mental health and resilience. The CSSS workshop was founded on the idea that schools can work to foster a sense of interpersonal connectedness and support that guards against the emergence of suicidality in young people. By guiding school personnel in the process of creating a roadmap for enhancing their schools’ suicide prevention and response initiatives, the CSSS training may help to move school staff closer to fulfilling their potential as agents of meaningful change in the mental health arena. Considering that states are increasingly requiring that suicide awareness and prevention training be provided to school employees, there is a heightened demand for training opportunities specifically tailored to the needs of school settings. As of February of 2018, 18 states plus the District of Columbia mandate suicide prevention training for school personnel, and 14 states encourage such instruction [43], though nationwide uniformity in the content and duration of programming has yet to be achieved [44]. The CSSS workshop may be of interest to school personnel seeking to satisfy their state’s training mandate or to school districts interested in assuming a more expansive role in suicide prevention. With appropriate guidance, schools can take their place among a tapestry of social institutions working to reduce the loss of young life to a preventable public health problem.

## Figures and Tables

**Table 1:** CSSS workshop participants’ pre-test and post-test ratings of items related to suicide safety in the schools (*N* = 562).

Item ^a^	Pre *M* (*SD*)	Post *M* (*SD*)	*df*	*t*	*p*
1. Youth suicide is a significant public health problem.	4.53 (0.68)	4.73 (0.56)	561	8.04	<.001 *
2. Schools should play an important role in youth suicide prevention.	4.61 (0.67)	4.77 (0.51)	561	5.99	<.001 *
3. My school has made suicide prevention a priority.	3.46 (0.96)	3.67 (0.95)	561	5.78	<.001 *
4. My school values efforts at suicide prevention.	3.92 (0.84)	4.00 (0.80)	561	2.26	.024 *
5. I have the knowledge I need to help a student at risk for suicide.	3.75 (0.88)	4.28 (0.59)	561	15.14	<.001 *
6. I have the knowledge I need to respond after a suicide death or attempt affects my school.	3.43 (0.94)	4.06 (0.67)	561	16.30	<.001 *
7. I know of some specific evidence-based suicide prevention programs for schools.	2.93 (0.98)	4.13 (0.71)	561	26.94	<.001 *
8. I know how to judge school-based suicide prevention/intervention programs against best practice standards.	2.76 (0.92)	3.82 (0.79)	561	26.19	<.001 *
9. I am familiar with some free or low-cost resources that could be used to enhance suicide safety at my school.	2.91 (1.00)	4.18 (0.68)	561	28.08	<.001 *
10. I am part of a team that is working to improve suicide safety at my school.	3.66 (1.01)	4.07 (0.87)	561	10.88	<.001 *
11. Our team has the support of our administration to work on improving suicide safety at our school.	3.91 (0.90)	4.08 (0.81)	561	4.84	<.001 *
12. I have the support I need to help a student at risk for suicide.	3.87 (0.82)	4.20 (0.66)	561	9.75	<.001 *
13. I have the support I need to respond after a suicide death or attempt affects my school.	3.64 (0.93)	4.07 (0.73)	561	11.48	<.001 *
14. My colleagues and I have a working action plan that delineates our next steps for improving suicide safety.	3.08 (1.00)	3.71 (0.93)	560	14.04	<.001 *
15. I feel empowered to work collaboratively with my colleagues on implementing our next steps for improving our school’s suicide safety.	3.93 (0.91)	4.40 (0.64)	560	12.32	<.001 *

Note. ^a^ Items were rated on a 5-point scale (1 = Strongly Disagree, 2 = Disagree, 3 = Neutral, 4 = Agree, 5 = Strongly Agree); * *p* ≤ 0.05.

**Table 2:** CSSS workshop participants’ pre-test and post-test composite scores (*N* = 562).

Area Assessed	Pre	Post	*df*	*t*	*p*	*d*
	*M* (*SD*)	*M* (*SD*)				
Attitudes ^a^						
All participants	9.14 (1.24)	9.51 (1.00)	561	7.94	<.001 *	0.47
With prior training	9.28 (1.06)	9.58 (1.01)	312	5.64	<.001 *	0.45
Without prior training	8.96 (1.42)	9.42 (0.99)	247	5.66	<.001 *	0.51
Attended alone	9.25 (1.14)	9.53 (1.00)	193	4.74	<.001 *	0.48
Attended with others	9.08 (1.30)	9.49 (1.02)	358	6.28	<.001 *	0.47
Knowledge ^b^						
All participants	15.79 (3.51)	20.47 (2.65)	561	32.87	<.001 *	1.96
With prior training	16.94 (3.34)	20.84 (2.62)	312	22.36	<.001 *	1.79
Without prior training	14.35 (3.18)	20.00 (2.63)	247	25.58	<.001 *	2.30
Attended alone	15.64 (3.77)	20.66 (2.62)	193	20.08	<.001 *	2.04
Attended with others	15.91 (3.38)	20.38 (2.69)	358	25.49	<.001 *	1.90
Support ^c^						
All participants	22.47 (4.02)	24.09 (3.71)	561	12.68	<.001 *	0.76
With prior training	23.06 (4.00)	24.43 (3.85)	312	8.85	<.001 *	0.71
Without prior training	21.69 (3.92)	23.62 (3.47)	247	9.12	<.001 *	0.82
Attended alone	22.01 (4.12)	23.57 (3.81)	193	7.17	<.001 *	0.73
Attended with others	22.75 (3.95)	24.42 (3.63)	358	10.40	<.001 *	0.78
Total Scale ^d^						
All participants	54.41 (8.10)	62.20 (6.89)	559	28.76	<.001 *	1.72
With prior training	56.52 (7.84)	63.14 (7.00)	312	21.12	<.001 *	1.69
Without prior training	51.70 (7.63)	60.98 (6.56)	245	20.44	<.001 *	1.84
Attended alone	53.68 (8.42)	61.69 (7.02)	191	17.59	<.001 *	1.79
Attended with others	54.88 (7.93)	62.56 (6.83)	358	22.37	<.001 *	1.67

* *p* ≤ 0.05; ^a^ Attitude composite is composed of items 1 and 2. Possible scores range from 2 to 10.; ^b^ Knowledge composite is composed of items 5, 6, 7, 8, and 9. Possible scores range from 5 to 25; ^c^ Support composite is composed of items 3, 4, 10, 11, 12, and 13. Possible scores range from 6 to 30; ^d^ total scale represents a summation of scores obtained for all items listed in Table 1. Scores range from 15 to 75.

**Table 3:** Participants’ satisfaction ratings of the CSSS workshop % (*n*).

Item	Strongly Disagree	Disagree	Neutral	Agree	Strongly Agree
1. The content of this workshop was relevant to my job.	0.2 (1)	0.0 (0)	1.1 (6)	18.5 (103)	80.3 (447)
2. I learned a lot from this workshop.	0.2 (1)	0.2 (1)	3.6 (20)	26.9 (150)	69.1 (385)
3. I found this workshop useful.	0.2 (1)	0.0 (0)	1.8 (10)	25.1 (140)	72.9 (406)
4. I would recommend this workshop to others.	0.2 (1)	0.0 (0)	2.2 (12)	23.9 (133)	73.8 (411)

**Table 4:** Ratings of school-based progress toward suicide safety at 3-month follow-up % (*n*).

Item	Strongly Disagree	Disagree	Neutral	Agree	Strongly Agree
1. The ideas and resources I brought back from the CSSS workshop were received positively	3.9 (2)	2.0 (1)	23.5 (12)	54.9 (28)	15.7 (8)
2. The ideas and resources I brought back from the CSSS workshop were received with enthusiasm	3.9 (2)	7.8 (4)	39.2 (20)	35.3 (18)	13.7 (7)
3. Since the CSSS workshop, our school has improved its suicide safety efforts	4.0 (2)	14.0 (7)	38.0 (19)	38.0 (19)	6.0 (3)
4. The CSSS workshop helped our school improve its suicide safety	3.9 (2)	7.8 (4)	35.3 (18)	43.1 (22)	9.8 (5)
5. Our school is doing a better job of responding to at-risk students than we were before the CSSS workshop	3.9 (2)	5.9 (3)	51.0 (26)	31.4 (16)	7.8 (4)
6. Our school is doing a better job of preventing suicide than we were before the CSSS workshop	3.9 (2)	9.8 (5)	51.0 (26)	29.4 (15)	5.9 (3)

Note. Data collected at 3-month follow-up (*n* = 51).

**Table 5:** Barriers to implementing school-based suicide prevention and suicide safety efforts at follow-up.

Barrier	% (*n*)
Not enough time	66.7 (32)
Stigma surrounding talking about suicide	27.1 (13)
School staff’s lack of confidence in the potential effectiveness of these efforts	16.7 (8)
Community resources/agencies are not responsive to school’s needs	16.7 (8)
School staff’s lack of knowledge/guidance as to how to proceed	14.6 (7)
Insufficient support from administrators	12.5 (6)
Staff members have difficulty working as a team	12.5 (6)
Poor communication with community resources/agencies	12.5 (6)
Inadequate funds	12.5 (6)
Insufficient parental support	10.4 (5)
Insufficient referral resources in the community	8.3 (4)

Note. Data collected at 3-month follow-up (*n* = 48).
